# Sleep Quality and Healthy Lifestyle Beliefs in Adolescents: The Mediating Role of Screen Exposure

**DOI:** 10.1002/brb3.71212

**Published:** 2026-03-10

**Authors:** Tuğba Solmaz, Mukaddes Demir Acar, Osman Demir

**Affiliations:** ^1^ Faculty of Health Sciences, Nursing Department Tokat Gaziosmapasa University Tokat Türkiye; ^2^ Faculty of Health Sciences, Department of Pediatric Nursing Tokat Gaziosmapasa University Tokat Türkiye; ^3^ Faculty of Medicine, Department of Biostatistics Tokat Gaziosmapasa University Tokat Türkiye

**Keywords:** adolescent, health behaviors, screen exposure, sleep quality

## Abstract

**Objective:**

Increased screen exposure among adolescents leads to short‐ or long‐term health problems. This study aimed to examine the mediating role of screen exposure in the relationship between sleep quality and healthy lifestyle among adolescents.

**Methods:**

This cross‐sectional and correlational study was conducted between February and May 2025 in a province in the Black Sea region of Turkey with 700 adolescents attending two high schools affiliated with the Provincial Directorate of National Education. Data were collected using the Descriptive Characteristics Form, the Richard–Campbell Sleep Questionnaire, the Adolescent Healthy Lifestyle Belief Scale, and the Screen Exposure of Adolescents (ESEA) Scale. Descriptive, Pearson correlation, regression, and mediation analyses were performed on the data.

**Results:**

Participants' screen exposure levels and healthy lifestyle beliefs were found to be moderate, while their sleep quality was good. Bootstrapping results revealed that screen exposure in adolescents led to a decrease in sleep quality (β = −0.091; *p* < 0.05). Increased screen exposure worsened healthy lifestyle beliefs (β = −0.327; *p* < 0.05). Sleep quality did not significantly affect healthy lifestyle beliefs (β = 0.061; *p* = 0.076). The study revealed that screen exposure played a mediating role in the relationship between sleep quality and healthy lifestyle (β = 0.030; 95% CI [0.008: 0.053]).

**Conclusion:**

It has been concluded that increased screen exposure affects sleep quality and beliefs about healthy lifestyles in adolescents. To improve sleep quality and healthy lifestyle beliefs among adolescents, it is necessary to reduce their screen exposure levels. Planning and implementing nursing interventions for this purpose is extremely important in terms of protecting and improving adolescent health.

## Graphical abstract

Screen exposure is becoming an increasingly significant problem among adolescents. Adolescents should devote more time to activities that support a healthy lifestyle. It has been demonstrated that screen exposure in this population significantly affects sleep quality and healthy lifestyle behaviors.

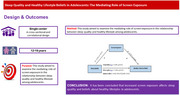

## 1 Introduction

The human brain is excessively stimulated and driven toward impulsive consumption, from television screens to massive billboards. Digital devices are widely used both on social media and for educational purposes, and adolescents constitute the majority of viewers in the virtual world. It has been reported that the average screen time spent in the digital world has increased in this population group (Majeed 2023; Zeyrek et al. 2024).

The time spent using a television, computer, tablet, or smartphone is defined as “screen exposure” (Schmidt et al. 2020). With the increasing prevalence of easily accessible mobile devices, numerous short‐ and long‐term health problems are emerging as a result of increased screen exposure among adolescents (Lavados‐Romo et al. 2023). Among these issues are unhealthy eating behaviors (Encarnação et al. 2023), lower quality of life (Lavados‐Romo et al. 2023), postural disorders (Mcallister et al. 2021), behavioral disorders (Encarnação et al. 2023), depressive symptoms (Glover et al. 2022), anxiety (Nguyen et al. 2022), hyperactivity (Vuriyanti et al. 2023), weaker self‐esteem (Glover et al. 2022), and poor sleep outcomes (Stiglic and Viner 2019).

Technologically advanced societies have seen poor sleep quality emerge as a significant public health issue, with increasing technology use contributing to the rise in sleep difficulties (Cheung and Wong 2011; Bilgrami et al. 2017; Maurya et al. 2022). Spending three or more hours per day in front of a screen has been associated with an increased risk of frequent sleep problems (Hysing et al. 2015). Problems in the sleep cycle have been reported to affect melatonin levels and increase the likelihood of mental health issues such as depression, anxiety, stress, and social dysfunction (Yogesh et al. 2014; Zeyrek et al. 2024). Sleep quality is of great importance in human life, both physiologically and socially, and significantly affects an individual's healthy lifestyle (Plaford 2009).

Adolescence is an important period during which the foundations for healthy lifestyle choices and behaviors are laid. Adolescents make independent decisions about healthy lifestyle behaviors, including physical activity and nutrition (Chen et al. 2014; Özdemir et al. 2020). Adolescents who turn health‐related behaviors into habits can maintain a healthier status, gain knowledge about health‐related issues, and improve their health status to a higher level (Fleary et al. 2018).

Investigating the relationship between sleep quality and healthy lifestyle in adolescents in relation to screen exposure is extremely important. Current studies have primarily addressed internet, technology, and social media addiction in adolescents (Shimoga et al. 2019; Infante and Mardikaningsih 2022) separately from healthy lifestyle and healthy living beliefs (Fleary et al. 2018; Akdeniz Kudubeş and Bektas 2020; Özdemir and Bülbül 2023), and have focused on how excessive use of online communication tools such as the internet addiction and smartphones affects sleep quality (Kurugodiyavar et al. 2018; Yücel and Ünsalver 2019; Coşkun and Kılıç 2022). However, the mediating role of screen exposure in the relationship between sleep quality and healthy lifestyle beliefs in this population has not been sufficiently investigated. Therefore, this study aims to comprehensively evaluate the relationship between screen exposure and sleep quality and healthy lifestyle beliefs among adolescents. By examining these relationships, the study will help identify new strategies to reduce screen exposure among adolescents and promote positive health behaviors.

### 1.1 Research Hypotheses

**H1_1_ **: There is a relationship between sleep quality and screen exposure in adolescents.
**H1_2_ **: There is a relationship between screen exposure and healthy lifestyle beliefs in adolescents.
**H1_3_ **: There is a relationship between sleep quality and beliefs about healthy lifestyles in adolescents.
**H1_4_ **: Screen exposure mediates the relationship between sleep quality and healthy lifestyle beliefs in adolescents.

## 2 Methods

### 2.1 Study Design and Participants

A cross‐sectional and correlational design was adopted. The study consisted of adolescents (aged 12–19) attending two high schools in a city center in the Black Sea region (n = 700). Schools were selected based on ease of access, choosing schools with similar socioeconomic levels. The sample size was calculated based on a previous study (Korkmaz and Tek Ayaz 2024) with a population size of 11,800, using a single‐sample design, 80% power, a 5% margin of error, and an effect size of 0.135, resulting in a minimum of 417. Considering that losses may occur during data collection, it was decided to include approximately 500 people in the study, which is 20% more than the sample size calculated. The study was completed with 700 adolescents. PASS version 15.0.5 was used for the sample size calculation (NCSS, LLC 2017). The inclusion criteria were: (1) willingness to participate in the study and (2) parental consent. The exclusion criterion was having a physical, cognitive, or psychological disability.

### 2.2 Data Collection Tools

The data were collected using the Descriptive Characteristics Form, the Screen Exposure of Adolescents (ESEA) Scale, the Richard—Campbell Sleep Questionnaire, and the Adolescent Healthy Lifestyle Belief Scale.

### 2.3 Descriptive Characteristics Form

The form was developed by researchers based on the literature and contained 16 questions covering students' descriptive characteristics such as age, gender, class, weight, height, family structure, income level, status of social security, parent's education status, parent's working status, sleep disorder status, etc. (Kurudirek et al. 2019; Sezer Efe et al. 2021; Coşkun and Kılıç 2022; Durak and Muslu 2023; Li et al. 2023; Gökçay et al. 2024; Korkmaz and Tek Ayaz 2024).

### 2.4 Screen Exposure of Adolescents (ESEA) Scale

Each sub‐dimension of the scale developed by Korkmaz and Tek Ayaz (2024) is evaluated independently. The total score evaluation of the scale indicates that an increase in the total score and the scores of the sub‐dimensions “willingness/desire for screen exposure, socialization, family control, procrastination tendency, and effects of prolonged screen exposure” indicates that the adolescent has a high level of screen exposure. In the family control sub‐dimension, the opposite is true. An increase in the score indicates that the adolescent's screen exposure is low. Scores range from 7 to 35 for the screen willingness/desire sub‐dimension, 3 to 15 for the socialization sub‐dimension, 3 to 15 for the family control sub‐dimension, 5 to 25 for the procrastination tendency sub‐dimension, and 5 to 25 for the effects of prolonged screen exposure sub‐dimension. The total scale score ranges from 23 to 115. Items 11, 12, and 13 on the scale are reverse‐scored. The Cronbach's alpha value of the scale is 0.79 (Korkmaz and Tek Ayaz 2024). In this study, the Cronbach's alpha value was found to be 0.78.

### 2.5 Richard—Campbell Sleep Questionnaire

The scale developed by Richards (1987) was validated and reliability tested in Türkiye (2015) by Karaman Özlü and Özer. The scale consists of 6 items that assess the depth of nighttime sleep, the time it takes to fall asleep, the frequency of waking up, the time spent awake after waking up, the quality of sleep, and the noise level in the environment. Each item is assessed on a scale from 0 to 100 using the visual analog scale technique. A score between “0 and 25” on the scale indicates very poor sleep, while a score between “76 and 100” indicates very good sleep. As the scale score increases, patients' sleep quality also improves. The Cronbach alpha value of the scale is 0.82 (Richards 1987; Karaman Özlü and Özer 2015). In this study, the Cronbach alpha value was found to be 0.77.

### 2.6 Adolescent Healthy Lifestyle Belief Scale

The scale was developed by Kelly et al. (2011) to assess beliefs related to a healthy lifestyle, and its Turkish adaptation study was conducted by Akdeniz Kudubeş and Bektas (2020). The scale emphasizes beliefs related to various aspects of maintaining a healthy lifestyle. The scale consists of 16 items and three sub‐dimension (health beliefs, physical activity, and nutrition). Items 4, 5, 6, 11, 12, 13, and 16 constitute the health beliefs sub‐dimension, items 2, 7, 9, 14, and 15 constitute the physical activity sub‐dimension, and items 1, 3, 8, and 10 constitute the nutrition sub‐dimension. The scale, prepared according to a five‐point Likert system, is answered on a scale of “1 = Strongly Disagree” to “5 = Strongly Agree.” A minimum of 16 and a maximum of 80 points can be obtained on the entire scale. A higher score on the scale indicates an increase in adolescents' belief in healthy living. The Cronbach's alpha value of the scale is 0.90 (Kelly et al. 2011; Akdeniz Kudubeş and Bektas 2020). In this study, Cronbach's alpha value was found to be 0.91.

### 2.7 Data Collection

The data were collected by researchers through face‐to‐face interviews between February and May 2025. Two separate interviews were conducted with students in the school environment. In the first interview, the purpose of the study was explained to the students, and then the parental consent form and voluntary participation form were distributed. Students were given two days to submit the forms. In the second interview, the forms were distributed to students who gave their consent, and the students filled them out independently under the researcher's supervision. The researchers also informed the students that there were no wrong answers on the forms and that they should mark all items on the scale completely. It took approximately 10–15 min to complete the questionnaires.

### 2.8 Data Analysis

Descriptive statistics such as frequency, percentage, mean, and standard deviation were used in the data evaluation of the study. Skewness and kurtosis values between +2 and ‐2 were considered as criteria for normality in the data analysis. The internal consistency coefficient was calculated using the Cronbach's alpha test. Pearson's correlation coefficient was used to determine the direction and strength of the linear relationship between quantitative variables. To determine whether screen exposure played a mediating role in the relationship between sleep quality and healthy lifestyle beliefs, regression‐based mediation analysis was performed using the bootstrap test. In all analyses, the significance level was set at *p* < 0.05 (IBM Corp. 2013).

### 2.9 Ethical Considerations

Ethics committee approval was received from the Social and Humanities Research Ethics Committee of Tokat Gaziosmanpaşa University (Decision no: 01/01‐58, Date: January 14, 2025), and institutional permission from the Tokat Provincial Directorate of National Education was obtained. Participants and their families were informed about the purpose of the study, and their written consent was obtained. The research was conducted according to the Declaration of Helsinki. Personal information was removed from the data to ensure anonymity, and the data obtained was protected by encryption on the researcher's computer.

## 3 Results

The average age of adolescents participating in the study was 15.81 ± 1.03 years, the average sleep duration was 7.39 ± 1.63 h, the average screen time was 4.29 ± 3.1 h (weekdays) and 6.02 ± 3.29 h (weekends), the average exercise duration was 1.44 ± 1.51 h (weekdays) and 1.65 ± 1.62 h (weekends), and the average body mass index value was 21.35 ± 3.4. It was determined that 60.7% of the participants were female, 35.1% were ninth grade students, 65.9% were of normal weight, and 73.7% experienced sleep disorders. 82.9% of the students came from nuclear families, 48.3% had sufficient income to cover their expenses, and 72.6% had health insurance. Of the participants' mothers, 39.9% had a high school education, and 38.3% of their fathers had a high school education. It was determined that 68.0% of the mothers did not work, and 92.0% of the fathers worked (Table 1).

**TABLE 1:** Descriptive characteristics of adolescents (N = 700).

Descriptive characteristics		Number (n)	Percentage (%)
Gender	Female	425	60.7
	Male	275	39.3
Grade	9th grade	246	35.1
	10th grade	188	26.9
	11th grade	209	29.9
	12th grade	57	8.1
BMI	< 18.5	139	19.9
	18.5–24.9	461	65.9
	≥ 25	100	14.3
Family structure	Nuclear family	580	82.9
	Extended family	86	12.3
	Divorced family	34	4.9
Income status	Income < expense	50	7.1
	Income = expense	338	48.3
	Income > expense	312	44.6
Status of social security	Yes	508	72.6
	No	192	27.4
Mother education status	Illiterate	6	0.9
	Literate	17	2.4
	Primary education	240	34.3
	High school	279	39.9
	University and above	158	22.6
Father education status	Illiterate	6	0.9
	Literate	11	1.6
	Primary education	126	18.0
	High school	268	38.3
	University and above	289	41.3
Mother working status	Working	224	32.0
	Not working	476	68.0
Father working status	Working	644	92.0
	Not working	56	8.0
Sleep disorder status	Yes	183	26.1
	No	184	26.3
	Sometimes	333	47.6
**Descriptive characteristics**		**Mean**	**SD**
Age (years)		15.81	1.03
BMI (kg/m^2^)		21.35	3.4
Sleep time (h)		7.39	1.63
Mean screen time, weekdays (h)		4.29	3.1
Mean screen time, weekends (h)		6.02	3.29
Daily exercise time, weekdays (h)		1.44	1.51
Daily exercise time, weekends (h)		1.65	1.62

**Abbreviations**: BMI = body mass index, h = hours, SD = standard deviation.

Table 2 presents the descriptive statistics obtained from the evaluation of the scales used in the study. The mean scores of participants on the sleep quality, screen exposure, and healthy lifestyle belief scales were found to be 62.05 ± 22.52, 62.42 ± 12.32, and 59.18 ± 12.57, respectively (Table 2).

**TABLE 2:** Distribution of Richard—Campbell Sleep Questionnaire, Screen Exposure Scale, and Adolescent Healthy Lifestyle Belief Scale scores.

Scales	Min.	Max.	Mean	SD
Richard – Campbell Sleep Questionnaire	0	100	62.05	22.52
Screen Exposure Scale	31	103	62.42	12.32
Adolescent Healthy Lifestyle Belief Scale	16	80	59.18	12.57

**Abbreviations**: Max: maximum; Min: minimum; SD = standard deviation

A Pearson correlation analysis was conducted to determine the relationships between screen exposure, sleep quality, and belief in a healthy lifestyle among adolescents, and the findings are presented in Table 3. A high level of negative (r = −0.091) and significant (*p* < 0.05) relationship was found between sleep quality and screen exposure among adolescents. The relationship between sleep quality and healthy lifestyle was found to be highly positive (r = 0.091) and significant (*p* < 0.05). The relationship between screen exposure and healthy lifestyle was found to be weakly negative (r = −0.032) and significant (*p* < 0.001) (Table 3).

**TABLE 3:** Correlation values of the study variables.

Scales		Sleep Quality	Screen Exposure	Healthy Lifestyle Belief
Sleep quality	r	1	−0.091	0.091
	*p*		**0.016**	**0.017**
	n	700	700	700
Screen exposure	r	−0.091	1	−0.332
	*p*	**0.000**		**<0.001**
	n	700	700	700
Healthy lifestyle belief	r	0.091	−0.332	1
	*p*	**0.017**	**<0.001**	
	n	700	700	700

*Note*: *Bold indicates important values, *p* < 0.05, r = Pearson correlation.

**FIGURE 1:**
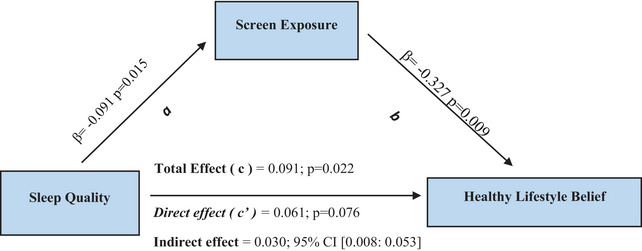
Model for the mediating role of screen exposure.

To determine whether screen exposure plays a mediating role in the relationship between sleep quality and belief in a healthy lifestyle, a bootstrapping analysis was performed, and the findings are presented in Table 4. The results showed that screen exposure was negatively related to sleep quality (β = −0.091; *p* < 0.05). An increase in screen exposure led to a decrease in healthy lifestyle (β = −0.327; *p* < 0.05). Sleep quality did not significantly affect healthy lifestyle (β = 0.061; *p* = 0.076). Analysis of the total effect excluding screen exposure, the mediating variable in the model, showed that the effect was statistically significant and positive (β = 0.091; *p* < 0.05). Screen exposure mediates the relationship between sleep quality and healthy lifestyle (β = 0.030; 95% CI [0.008: 0.053]) (Table 4; Figure 1).

**TABLE 4:** Path coefficients for direct, indirect and total effects of the model.

Path Coefficient	β	p	%95 confidence	
			Interval lower limit	Upper limit
Sleep quality ‐ screen exposure (direct effect a)	−0.091	**0.015**	−0.170	−0.025
Screen exposure ‐ healthy lifestyle belief (direct effect‐b)	−0.327	**0.009**	−0.398	−0.267
Sleep quality ‐ healthy lifestyle belief (direct effect‐c’)	0.061	0.076	−0.005	0.118
Sleep quality ‐ healthy lifestyle belief (indirect effect a.b)	0.030	**0.020**	0.008	0.053
Sleep quality ‐ healthy lifestyle belief (total effect‐ c)	0.091	**0.022**	0.025	0.153

*Note*: *Bold indicates important values, *p* <  0.05, β = unstandardized regression coefficient.

## 4 Discussion

Before starting the discussion, we would like to briefly summarize the main findings of the study. Sleep quality was associated with beliefs about healthy lifestyles. In line with our study hypotheses, we found that increased screen exposure in adolescents leads to a decline in sleep quality and worsens beliefs about healthy lifestyles. Furthermore, we concluded that screen exposure plays a mediating role in the relationship between sleep quality and beliefs about healthy lifestyles.

Adolescence is a special period characterized by a certain level of self‐definition and self‐uncertainty, along with numerous changes in cognition, emotions, roles, and behavior (Kuang et al. 2023a). Under varying psychological conditions, adolescents may explore and develop different lifestyles, and the likelihood of deviating from a healthy lifestyle may be higher during this period. It also leads to problems such as low self‐control, cell phone or screen addiction, weaker health beliefs, and lower levels of physical activity (Kuang et al. 2023b). A study conducted in China reports that students' health beliefs improve their sleep quality and that physical activity also increases sleep quality (Gao et al. 2023). Current studies reveal that sleep quality is closely related to physical activity, mindfulness, health literacy, and belief in a healthy lifestyle (Ji et al. 2022, Mirolli et al. 2021, Ono et al. 2021). Adequate sleep duration seems to be highly influenced by factors related to individual lifestyles, family and school eating behaviors, and diet quality (Godos et al. 2025). Our study suggests that health‐protective and health‐promoting activities aimed at increasing healthy lifestyles among adolescents are very important and are also closely related to sleep quality.

Our study found that adolescents had a moderate average screen exposure score; they were exposed to screens for 4.29 ± 3.1 h on weekdays and 6.02 ± 3.29 h on weekends. According to data reported by parents in the 2016 National Children's Health Survey, every hour spent in front of a digital screen was associated with a 3–8 min decrease in sleep duration on weeknights and more irregular bedtimes. This indicates that 5 h of screen time per day can reduce sleep duration by 40 min or more, while 10 h of screen time per day can result in 80 min less sleep (Przybylski 2019). In a study conducted by Shochat et al. (2010), difficulty falling asleep is a common problem among adolescents, and exposure to electronic screens may have stimulating effects (Shochat et al. 2010). With the proliferation of the internet and smartphones, mobile phone addiction has become a significant factor triggering sleep disorders (Cui et al. 2021).

A study has reported that excessive screen time and its effects, while varying depending on the type of screen activity, have been identified as a significant risk factor for poor sleep, and that excessive social media use is associated with sleep deprivation (χ2 = 8.1726; *p* < 0.001) (Zhong et al. 2025). Another study reported that internet‐addicted students had significantly shorter sleep durations and delayed bedtimes compared to those with less screen time (Kawabe et al. 2019). A study conducted with adolescents in Sweden found that nighttime messaging is a significant factor contributing to delayed bedtimes, sleep deprivation, daytime fatigue, and disrupted sleep patterns (Garmy and Ward 2018). A study by Chang et al. (2015) shows that using light‐emitting electronic devices in the hours before bedtime can have a significant effect on sleep, wakefulness, and the circadian clock (Chang et al. 2015). Another study concluded that increased screen exposure among adolescents is associated with sleep problems, mental health issues (Santos et al. 2023), and physical problems such as myopia (Zong et al. 2024; De Sá et al. 2023). Similar to the literature, this study also found that the relationship between sleep quality and screen exposure in adolescents was highly negative (r = −0.091) and significant (*p* < 0.05). Therefore, parents and policymakers should consider taking important strategic steps to improve sleep quality in adolescents.

This study demonstrates that increased screen exposure worsens healthy lifestyle habits (β = −0.327; *p* < 0.05). One study found that cell phone addiction was associated with a significant reduction in the effect size of health belief on sleep quality, the effect size of health belief on physical activity, and the effect size of physical activity on sleep quality (Gao et al. 2023). These findings raise significant public health concerns regarding adolescents' lifestyle and sleep quality. Parents should take steps to reduce screen exposure in adolescents, and the physical and mental health of today's youth, who are shaping the future of a healthy society, should be protected. In this context, the development of national and international policies is recommended. Future researchers are encouraged to design experimental studies to evaluate the relationships and potential causes between increasing screen exposure, sleep outcomes, and healthy lifestyle factors in adolescents.

### 4.1 Limitations

The limitations of this study include the fact that it was conducted in two high schools in a province located in Türkiye's Black Sea Region and that it relied on self‐reported data from adolescents.

### 4.2 Implications to Practice

Our study has revealed that the increasing screen exposure among adolescents negatively impacts sleep quality and beliefs about healthy lifestyles, highlighting the urgent need for preventive public health initiatives. For example, since increased screen time is particularly associated with a decrease in sleep quality and healthy lifestyle behaviors, public health campaigns that collaborate with adolescents and their families in schools to direct adolescents toward sports or artistic activities that protect them from screen exposure may be beneficial. At the same time, it is thought that the existing evidence could guide the integration of healthy lifestyle behaviors into school‐based health education curricula and the development of age‐appropriate screen time guidelines in schools by policymakers.

## 5 Conclusion

In conclusion, this study emphasizes that increased screen exposure among adolescents negatively impacts sleep quality and healthy lifestyle habits. It also highlights the need for interventions promoting digital hygiene to establish healthier sleep routines and healthy lifestyle behaviors among adolescents. Accordingly, it is considered extremely important to plan interventions aimed at reducing screen exposure among adolescents, increasing social interactions, and developing alternative activities. Accordingly, it is recommended to promote interventions aimed at reducing screen exposure, increasing social interaction, and developing alternative activities among adolescents. Implementing these recommendations may help adolescents adopt healthier lifestyles, regulate their sleep quality, and reduce their screen exposure. Furthermore, experimental studies can be designed in the future to test the effectiveness of interventions aimed at reducing screen exposure among adolescents.

## Author Contributions

**Tuğba Solmaz**: conceptualization, investigation, writing – original draft, methodology, writing – review and editing, project administration. **Mukaddes Demir Acar**: investigation, writing – original draft, methodology, writing – review and editing, supervision. **Osman Demir**: data curation, formal analysis, supervision. AI was used to improve the language and edit of the text.

## Funding

The authors have nothing to report.

## Conflicts of Interest

The authors declare no conflicts of interest.

## Ethics Statement

Ethics committee approval was received from the Social and Humanities Research Ethics Committee of Tokat Gaziosmanpaşa University (Decision no: 01/01‐58, Date: January 14, 2025) and institutional permission from the Tokat Provincial Directorate of National Education were obtained. Participants and their families were informed about the purpose of the study, and their written consent was obtained. The research was conducted according to the Declaration of Helsinki.

## Data Availability

The data are available on reasonable request from the corresponding author. The data are not publicly available due to privacy or ethical restrictions.

## References

[brb371212-bib-0001] Akdeniz Kudubeş, A. , and M. Bektas . 2020. “Orginal Article: Psychometric Properties of the Turkish Version of the Healthy Lifestyle Belief Scale for Adolescents.” Journal of Pediatric Nursing 53: e57–e63. 10.1016/j.pedn.2020.02.006. 32139234

[brb371212-bib-0002] Bilgrami, Z. , L. McLaughlin , R. Milanaik , and A. Adesman . 2017. “Health İmplications of New‐Age Technologies: A Systematic Review.” Minerva Pediatrica 69, no. 4: 348–367. 10.23736/s0026-4946.17.04937-4. 28425691

[brb371212-bib-0003] Chang, A. M. , D. Aeschbach , J. F. Duffy , and C. A. Czeisler . 2015. “Evening Use of Light‐Emitting Ereaders Negatively Affects Sleep, Circadian Timing, and Next‐Morning Alertness.” Proceedings of the National Academy of Sciences 112, no. 4: 1232–1237. 10.1073/pnas.1418490112. PMC4313820 25535358

[brb371212-bib-0004] Chen, M. Y. , L. J. Lai , H. C. Chen , and J. Gaete . 2014. “Development and Validation of the Short‐Form Adolescent Health Promotion Scale.” BMC Public Health [Electronic Resource] 14: 1106. 10.1186/1471-2458-14-1106. 25344693 PMC4216378

[brb371212-bib-0005] Cheung, L. M. , and W. S. Wong . 2011. “The Effects of Insomnia and Internet Addiction on Depression in Hong Kong Chinese Adolescents: an Exploratory Cross‐Sectional Analysis.” Journal of Sleep Research 20, no. 2: 311–317. 10.1111/j.1365-2869.2010.00883.x. 20819144

[brb371212-bib-0006] Coşkun, S. , and H. S. Kılıç . 2022. “Ergenlerin İnternet Ve Akıllı Telefon Bağımlılığının Yalnızlık, Uyku Kalitesi Ve Akademik Başarı Düzeyleri Ile İlişkisi.” Bağımlılık Dergisi 23, no. 4: 511–521. 10.51982/bagimli.1097365.

[brb371212-bib-0007] Cui, G. , Y. Yin , S. Li , et al. 2021. “Longitudinal Relationships Among Problematic Mobile Phone Use, Bedtime Procrastination, Sleep Quality and Depressive Symptoms in Chinese College Students: A Cross‐Lagged Panel Analysis.” BMC Psychiatry [Electronic Resource] 21, no. 1: 449. 10.1186/s12888-021-03451-4. 34507561 PMC8431882

[brb371212-bib-0008] De Sá, S. , A. Baião , H. Marques , et al. 2023. “The Influence of Smartphones on Adolescent Sleep: A Systematic Literature Review.” Nursing Reports 13, no. 2: 612–621. 10.3390/nursrep13020054. 37092482 PMC10123719

[brb371212-bib-0009] Durak, Y. , and G. K. Muslu . 2023. “Adölesanların Sağlıklı Yaşam Tarzı İnançları ile Sürdürülebilir Yaşama Yönelik Farkındalıkları Arasındaki İlişkinin İncelenmesi.” Unika Sağlık Bilimleri Dergisi 3, no. 3: 588–600.

[brb371212-bib-0010] Encarnação, S. , F. Rodrigues , A. M. Monteiro , et al. 2023. “Obesity Status and Physical Fitness Levels in Male and Female Portuguese Adolescents: A Two‐Way Multivariate Analysis.” International Journal of Environmental Research and Public Health 20, no. 12: 6115. 10.3390/ijerph20126115. 37372702 PMC10298555

[brb371212-bib-0011] Fleary, S. A. , P. Joseph , and J. E. Pappagianopoulos . 2018. “Adolescent Health Literacy and Health Behaviors: A Systematic Review.” Journal of Adolescence 62: 116–127. 10.1016/j.adolescence.2017.11.010. 29179126

[brb371212-bib-0012] Gao, X. , C. Li , B. Han , P. Xu , and C. Qu . 2023. “The Relationship Between Health Belief and Sleep Quality of Chinese College Students: The Mediating Role of Physical Activity and Moderating Effect of Mobile Phone Addiction.” Frontiers in Public Health 11: 1108911. 10.3389/fpubh.2023.1108911. 37124819 PMC10133522

[brb371212-bib-0013] Garmy, P. , and T. M. Ward . 2018. “Sleep Habits and Nighttime Texting Among Adolescents.” The Journal of School Nursing 34, no. 2: 121–127. 10.1177/1059840517704964. 28421911

[brb371212-bib-0014] Glover, J. , M. Ariefdjohan , and S. L. Fritsch . 2022. “# KidsAnxiety and the Digital World.” Child and Adolescent Psychiatric Clinics 31, no. 1: 71–90. 10.1016/j.chc.2021.06.004. 34801156

[brb371212-bib-0015] Godos, J. , A. Rosi , F. Scazzina , et al. 2025. “Diet, Eating Habits, and Lifestyle Factors Associated with Adequate Sleep Duration in Children and Adolescents Living in 5 Mediterranean Countries: The DELICIOUS Project.” Nutrients 17, no. 7: 1242. 10.3390/nu17071242. 40218999 PMC11990884

[brb371212-bib-0016] Gökçay, G. , S. E. Eryilmaz , and F. Küçük . 2024. “The Impact of Social Media Addiction on Healthy Lifestyle Beliefs in Adolescents.” Journal of Pediatric Nursing 76: e85–e92. 10.1016/j.pedn.2024.01.023. 38307755

[brb371212-bib-0017] Hysing, M. , S. Pallesen , K. M. Stormark , R. Jakobsen , A. J. Lundervold , and B. Sivertsen . 2015. “Sleep and Use of Electronic Devices in Adolescence: Results From a Large Population‐Based Study.” BMJ Open 5, no. 1: e006748. 10.1136/bmjopen-2014-006748. PMC4316480 25643702

[brb371212-bib-0018] IBM Corp . 2013. IBM SPSS Statistics for Windows, Version 22.0 . IBM Corp.

[brb371212-bib-0019] Infante, A. , and R. Mardikaningsih . 2022. “The Potential of Social Media as a Means of Online Business Promotion.” Journal of Social Science Studies 2, no. 2: 45–49.

[brb371212-bib-0020] Ji, C. , J. Yang , L. Lin , and S. Chen . 2022. “Physical Exercise Ameliorates Anxiety, Depression and Sleep Quality in College Students: Experimental Evidence from Exercise Intensity and Frequency.” Behavioral Sciences 12, no. 3: 61. 10.3390/bs12030061. 35323380 PMC8944991

[brb371212-bib-0021] Karaman Özlü, Z. , and N. Özer . 2015. “Richard‐Campbell Sleep Questionnaire Validity and Reliability Study.” Journal of Turkish Sleep Medicine 2, no. 2: 29–32. 10.4274/jtsm.02.008.

[brb371212-bib-0022] Kawabe, K. , F. Horiuchi , Y. Oka , and S. I. Ueno . 2019. “Association Between Sleep Habits and Problems and Internet Addiction in Adolescents.” Psychiatry Investigation 16, no. 8: 581–587. 10.30773/pi.2019.03.21.2. 31389226 PMC6710414

[brb371212-bib-0023] Kelly, S. A. , B. M. Melnyk , D. L. Jacobson , and J. A. O'Haver . 2011. “Correlates Among Healthy Lifestyle Cognitive Beliefs, Healthy Lifestyle Choices, Social Support, and Healthy Behaviors in Adolescents: Implications for Behavioral Change Strategies aand Future Research.” Journal of Pediatric Health Care 25, no. 4: 216–223. 10.1016/j.pedhc.2010.03.002. 21700136

[brb371212-bib-0024] Korkmaz, M. , and S. Tek Ayaz . 2024. “Development of the Evaluating the Screen Exposure of Adolescents (ESEA) Scale: A Validation and Reliability Study.” Journal of Pediatric Nursing 78: e213–e218. 10.1016/j.pedn.2024.07.008. 39019738

[brb371212-bib-0025] Kuang, J. , J. J. Arnett , E. Chen , et al. 2023b. “Examining Behavioral Problems Among Chinese Emerging Adults: The Mediating Role of Physical Activity and Self‐Control.” International Journal of Mental Health Promotion 25: 1–14. 10.32604/ijmhp.2023.029187.

[brb371212-bib-0026] Kuang, J. , J. Zhong , P. Yang , et al. 2023a. “Psychometric Evaluation of the Inventory of Dimensions of Emerging Adulthood (IDEA) in China.” International Journal of Clinical and Health Psychology 23, no. 1: 100331. 10.1016/j.ijchp.2022.100331. 36247406 PMC9529670

[brb371212-bib-0027] Kurudirek, F. , N. Gürarslan Baş , and D. Arıkan . 2019. “The Relationship between Sleep Quality and Smartphone Addiction among Adolescents.” University of Health Sciences Journal of Nursing 6, no. 2: 117–124. 10.48071/sbuhemsirelik.1385723.

[brb371212-bib-0028] Kurugodiyavar, M. D. , H. R. Sushma , M. Godbole , and M. S. Nekar . 2018. “Impact of Smartphone Use on Quality of Sleep Among Medical Students.” International Journal of Community Medicine and Public Health 5, no. 1: 101. 10.18203/2394-6040.ijcmph20175604.

[brb371212-bib-0029] Lavados‐Romo, P. , O. Andrade‐Mayorga , G. Morales , S. Muñoz , and T. Balboa‐Castillo . 2023. “Association of Screen Time and Physical Activity with Health‐Related Quality of Life in College Students.” Journal of American College Health 71, no. 5: 1504–1509. 10.1080/07448481.2021.1942006. 34242535

[brb371212-bib-0030] Li, J. , X. Zhou , Z. Huang , and T. Shao . 2023. “Effect of Exercise Intervention on Depression in Children and Adolescents: A Systematic Review and Network Meta‐Analysis.” BMC Public Health [Electronic Resource] 23, no. 1: 1918. 10.1186/s12889-023-16824-z. 37794338 PMC10552327

[brb371212-bib-0031] Majeed, S. 2023. “Screen Time and Aggression in Adolescents: A Moderating Role of Content‐Based Media Exposure.” Journal of Pakistan Psychiatric Society 20, no. 3: 15–21. 10.63050/jpps.20.03.204.

[brb371212-bib-0032] Maurya, C. , T. Muhammad , P. Maurya , and P. Dhillon . 2022. “The Association of Smartphone Screen Time With Sleep Problems Among Adolescents and Young Adults: Cross‐Sectional Findings From India.” BMC Public Health [Electronic Resource] 22, no. 1: 1686. 10.1186/s12889-022-14076-x. 36064373 PMC9444278

[brb371212-bib-0033] Mcallister, C. , G. C. Hisler , A. B. Blake , J. M. Twenge , E. Farley , and J. L. Hamilton . 2021. “Associations Between Adolescent Depression and Self‐Harm Behaviors and Screen Media Use in a Nationally Representative Time‐Diary Study.” Research on Child and Adolescent Psychopathology 49, no. 12: 1623–1634. 10.1007/s10802-021-00832-x. 34297316

[brb371212-bib-0034] Mirolli, M. , L. Simione , M. Martoni , and M. Fabbri . 2021. “Accept Anxiety to Improve Sleep: The Impact of the COVID‐19 Lockdown on the Relationships Between Mindfulness, Distress, and Sleep Quality.” International Journal of Environmental Research and Public Health 18, no. 24: 13149. 10.3390/ijerph182413149. 34948759 PMC8701850

[brb371212-bib-0035] NCSS, LLC . 2017. PASS 15 Power Analysis and Sample Size Software (Version 15.0.5) . NCSS.

[brb371212-bib-0036] Nguyen, C. T. T. , H.‐J. Yang , G. T. Lee , L. T. K. Nguyen , and S.‐Y.u Kuo . 2022. “Relationships of Excessive Internet Use with Depression, Anxiety, and Sleep Quality Among High School Students in Northern Vietnam.” Journal of Pediatric Nursing 62: e91–e97. 10.1016/j.pedn.2021.07.019. 34334256

[brb371212-bib-0037] Ono, S. , H. Ogi , M. Ogawa , D. Nakamura , T. Nakamura , and K. P. Izawa . 2021. “Relationship Between Parents' Health Literacy and Children's Sleep Problems in Japan.” BMC Public Health [Electronic Resource] 21, no. 1: 791. 10.1186/s12889-021-10864-z. 33894754 PMC8070322

[brb371212-bib-0038] Özdemir, S. , and F. Bülbül . 2023. “Adölesanlarda Sağlığı Geliştirme Ve Yaşamda Anlam Arasındaki İlişki.” Hacettepe Üniversitesi Hemşirelik Fakültesi Dergisi 10, no. 1: 1–8. 10.31125/hunhemsire.1272589.

[brb371212-bib-0039] Özdemir, S. , F. Küçük , S. Balcı , and A. Türköz . 2020. “11‐18 Yaş Arasındaki Adolesanların İnternet Bağımlılık Düzeyleri.” Balıkesir Sağlık Bilimleri Dergisi 9, no. 2: 83–92.

[brb371212-bib-0040] Plaford, G. R. 2009. Sleep and Learning: The Magic That Makes Us Healthy and Smart . R&L Education.

[brb371212-bib-0041] Przybylski, A. K. 2019. “Digital Screen Time and Pediatric Sleep: Evidence From a Preregistered Cohort Study.” The Journal of Pediatrics 205: 218–223.e1. 10.1016/j.jpeds.2018.09.054. 30396683

[brb371212-bib-0042] Richards, K. 1987. “Techniques for Measurement of Sleep in Critical Care.” Focus on Critical Care 14, no. 14: 34–40. 3650169

[brb371212-bib-0043] Santos, R. M. S. , C. G. Mendes , G. Y. Sen Bressani , et al. 2023. “The Associations Between Screen Time and Mental Health in Adolescents: A Systematic Review.” BMC Psychology 11, no. 1: 127. 10.1186/s40359-023-01166-7. 37081557 PMC10117262

[brb371212-bib-0044] Schmidt, S. C. E. , B. Anedda , A. Burchartz , et al. 2020. “Physical Activity and Screen Time of Children and Adolescents before and during the COVID‐19 Lockdown in Germany: A Natural Experiment.” ScientificReports 10, no. 1: 21780. 10.1038/s41598-020-78438-4. PMC7733438 33311526

[brb371212-bib-0045] Sezer Efe, Y. , E. Erdem , and B. Vural . 2021. “Lise Öğrencilerinde Siber Zorbalık Ve İnternet Bağımlılığı.” Bağımlılık Dergisi 22, no. 4: 465–473. 10.51982/bagimli.936930.

[brb371212-bib-0046] Shimoga, S. V. , E. Erlyana , and V. Rebello . 2019. “Associations of Social Media Use With Physical Activity and Sleep Adequacy among Adolescents: Cross‐Sectional Survey.” Journal of Medical Internet Research [Electronic Resource] 21, no. 6: e14290. 10.2196/14290. 31215512 PMC6604510

[brb371212-bib-0047] Shochat, T. , O. Flint‐Bretler , and O. Tzischinsky . 2010. “Sleep Patterns, Electronic Media Exposure and Daytime Sleep‐Related Behaviours among Israeli Adolescents.” Acta Paediatrica 99, no. 9: 1396–1400. 10.1111/j.1651-2227.2010.01821.x. 20377536

[brb371212-bib-0048] Stiglic, N. , and R. M. Viner . 2019. “Effects of Screentime on the Health and Well‐Being of Children and Adolescents: A Systematic Review of Reviews.” BMJ Open 9, no. 1: e023191. 10.1136/bmjopen-2018-023191. PMC6326346 30606703

[brb371212-bib-0049] Vuriyanti, M. , I. Rahmawati , and S. Suhari . 2023. “The Effect of Gadjet on Attention Deficit Hyperactivity Disorder (ADHD) in Preschool Children: Literature Review.” In UNEJ e‐Proceeding 287–293.

[brb371212-bib-0050] Yogesh, S. , S. Abha , and S. Priyanka . 2014. “Short Communication Mobile Usage and Sleep Patterns Among Medical Students.” Indian Journal of Physiology and Pharmacology 58, no. 1: 100–103. 25464686

[brb371212-bib-0051] Yücel, E. E. , and B. Ö. Ünsalver . 2019. “The Relationship Between Smartphone Using Style and Sleep Quality and Psychiatric Symptoms Among a Foundation University Students.” The European Research Journal 6, no. 6: 569–579. 10.18621/eurj.538377.

[brb371212-bib-0052] Zeyrek, I. , M. F. Tabara , and M. Çakan . 2024. “Exploring the Relationship of Smartphone Addiction on Attention Deficit, Hyperactivity Symptoms, and Sleep Quality Among University Students: A Cross‐Sectional Study.” Brain and Behavior 14, no. 11: e70137. 10.1002/brb3.70137. 39576227 PMC11583478

[brb371212-bib-0053] Zhong, C. C. , M. Chen , Z. Li , et al. 2025. “Examining Sleep Habits and Associated Lifestyle Factors in Adolescents: A Population‐Based Study.” Brain and Behavior 15, no. 10: e70885. 10.1002/brb3.70885. 41066770 PMC12510639

[brb371212-bib-0054] Zong, Z. , Y. Zhang , J. Qiao , Y. Tian , and S. Xu . 2024. “The Association Between Screen Time Exposure and Myopia in Children and Adolescents: A Meta‐Analysis.” BMC Public Health [Electronic Resource] 24, no. 1: 1625. 10.1186/s12889-024-19113-5. 38890613 PMC11186094

